# Commentary: Lower Urinary Tract Symptoms and Sexual Dysfunction: A Systematic Review and Meta-Analysis

**DOI:** 10.3389/fmed.2021.716371

**Published:** 2021-08-11

**Authors:** Tariq F. Al-Shaiji

**Affiliations:** Urology Unit, Department of Surgery, Jaber Al-Ahmad Al-Jaber Al-Sabah Hospital, Kuwait City, Kuwait

**Keywords:** lower urinary track symptoms, erectile dysfunction, prescription drugs, phosphodiesterase type 5 inhibitor, ethics–clinical

I read with interest a recently published article by Song et al. entitled “Lower Urinary Tract Symptoms and Sexual Dysfunction: A Systematic Review and Meta-Analysis” (1). The authors rightfully demonstrated an association between the presence of lower urinary tract symptoms (LUTS) and risk of sexual dysfunction. They pointed out the importance of sexual assessment in men presenting with LUTS. This comes with no surprise since this association is well-established and supported by the literature. Kirby et al. nicely summarized this relationship with epidemiological evidence from several community studies (2). In addition, a study in a population of 1,267 men presenting for prostate cancer screening confirmed that LUTS is an age-independent predictor of erectile dysfunction (ED) and that not only the presence of LUTS increases the likelihood of developing ED, but its severity is associated positively with the intensity of ED (3). Therefore, given that LUTS and ED are correlated, it is recommended that men seeking consultation for one condition should always be screened for complaints about the other condition (2). Song et al. also showed that the severity of LUTS was associated with not just ED, but with intercourse satisfaction, and overall satisfaction as well-except for sexual desire (1). In general, this is a nice meta-analysis summarizing multiple studies; however, the authors briefly touched on the available medications that can treat both conditions which I would like to elaborate on since the prescribing method for these medications deserves special attention that should not be overlooked.

This intimate relationship between LUTS and ED has open doors for therapeutic options that can treat both conditions simultaneously. One of these options has been phosphodiesterase type-5 inhibitors (PDE-5I), specifically a daily dosing with tadalafil 5 mg. The drug is currently approved by the Food and Drug Administration (FDA) to treat ED, the signs, and symptoms of benign prostatic hyperplasia (BPH), and ED and the signs and symptoms of BPH (ED/BPH) when the conditions coincide. PDE-5I are well-established modalities in the management of ED and considered first line. The daily dosing was found to be safe in the treatment of LUTS in the presence of ED and that it prolongs the ejaculatory latency time (4). Furthermore, tadalafil 5 mg daily treatment as monotherapy or in combination with α-blockers have shown efficacy in treating both LUTS and ED. Sebastianelli et al. indicated that tadalafil 5 mg should be considered a primary treatment option for patients with LUTS/BPH and ED with an excellent tolerability, safety, and effectiveness profile, both alone or in combination with tamsulosin 0.4 mg (5). Tadalafil is not just a treatment alternative to other established drugs for LUTS that have some sexual adverse events associated with their use, it is the only drug that can treat both ED and LUTS at the same time (6).

Having highlighted the former points, I have some concerns that I would like to point out. Although the data concerning tadalafil in the management of ED is firm, almost all health authorities worldwide whether being governmental, private, or insurance do not cover the treatment for this indication. This means that the patient must purchase the drug from his own pocket. In contrast, if the indication for its use is LUTS alone or LUTS/ED then coverage is available. This is never simple since many physicians, deliberately or not, prescribe the drug for a presumed indication of LUTS or LUTS/ED when the real indication is ED in the absence of LUTS especially in young men. This of course could be driven by patient's request or industry driven. In addition, patients seem to know the “trick” by claiming to suffer from LUTS or LUTS/ED when the real reason is purely ED or recreational use. This attitude of prescribing may lead to a negative effect on the heath system and health audit which is beyond the scope of this editorial. In essence, I recommend that further exploration should be carried out to determine the best way to monitor the way we prescribe this medication when ED and LUTS are concerned to overcome this ethical dilemma.

## Author Contributions

TA-S drafted and revised the article critically for important intellectual content and approved the final version to be published.

## Conflict of Interest

The author declares that the research was conducted in the absence of any commercial or financial relationships that could be construed as a potential conflict of interest.

## Publisher's Note

All claims expressed in this article are solely those of the authors and do not necessarily represent those of their affiliated organizations, or those of the publisher, the editors and the reviewers. Any product that may be evaluated in this article, or claim that may be made by its manufacturer, is not guaranteed or endorsed by the publisher.
